# Postscript: The future of the Greenland Ecosystem Monitoring programme

**DOI:** 10.1007/s13280-016-0871-9

**Published:** 2017-01-23

**Authors:** Torben R. Christensen, Elmer Topp-Jørgensen, Mikael K. Sejr, Niels Martin Schmidt

**Affiliations:** 1 Department of Earth and Ecosystem Science, Lund University, Sölvegatan 12, 22362 Lund, Sweden; 2 Department of Bioscience, Arctic Research Centre, Aarhus University, Frederiksborgvej 399, 4000 Roskilde, Denmark; 3 Department of Bioscience, Aarhus University, Vejlsøvej 25, building A2.11, 8600 Silkeborg, Denmark; 4 Arctic Research Centre, Aarhus University, Ny Munkegade bldg 1540, 8000 Aarhus, Denmark

As documented with examples in this special issue of *Ambio*, the Greenland Ecosystem Monitoring (GEM) Programme in its first 20 years has focused on both detailed and very comprehensive studies of specific ecosystems in order to monitor and understand ecosystem patterns and processes. An international review of GEM conducted in 2015 praised the programme for its comprehensiveness, but also saw an untapped potential for a wider use of GEM data and results. The review provided key recommendations for the future development of GEM that together with input from government bodies and internal scientific development considerations has led to the development of a new 5-year strategy for GEM (2017–2021).

## The GEM Strategy 2017–2021

### The rationale of GEM

Greenland Ecosystem Monitoring is a long-term integrated monitoring and research programme on ecosystems and climate change effects and feedbacks in the Arctic. The programme has established a coherent and integrated understanding of the functioning of ecosystems in a highly variable climate, which is based upon a comprehensive, long-term interdisciplinary data collection.

The major strategic strength of GEM is its scientifically integrated approach to the study of ecosystems based on concurrent long-term collection of data on climate, landscape processes, geophysics, biology and biogeochemistry in the marine, terrestrial, limnic and glaciological compartments of the ecosystem (see Fig. 1 in Christensen et al. 2017) across a climatic gradient from High- to Low-Arctic regions of Greenland. This provides a unique foundation for mapping and analysing ecosystem responses to temporary and more permanent climate changes within specific and different climatic regimes. This approach also improves the understanding of feedbacks between arctic ecosystems and the global climate system.

The GEM 2017–2021 strategy will further integrate the thematic sub-programmes through interdisciplinary initiatives answering key scientific questions on an ecosystem and Greenlandic scale.

#### Key overarching science questions addressed by the GEM strategy 2017–2021

- What are the principal connections between the cryosphere, freshwater, land and near coastal processes in Greenland and how do they vary in time and space?
- What are the implications of climate change and variability for ecosystem processes in Greenland?

### Consolidating existing research and monitoring

The five GEM sub-programmes (Climate Basis, GeoBasis, BioBasis, MarineBasis and GlacioBasis) are led by specialised research teams represented in international scientific networks and organisations. Participation in international initiatives is paramount to GEM as a mean to influence and implement international standards and science agendas. The priority is to maintain integrity of ongoing core GEM monitoring and continue efforts to standardise methodologies across sub-programmes, and develop smarter data acquisition and analysis through for instance automated measurements and recognition techniques where applicable. While continuing the existing time series, GEM must continue to be flexible in order to facilitate adaptive monitoring and address potential changes in science agendas and policy needs.

The GEM sub-programmes will continue to integrate external research projects on key ecosystem processes of relevance to GEM. Following the new strategy, GEM will further integrate the GEM sub-programmes through interdisciplinary initiatives answering key scientific questions on an ecosystem and Greenlandic scale.

### Upscaling and prediction

The comprehensive knowledge of climate change and ecosystem processes generated at GEM main sites constitutes an untapped potential for upscaling and prediction through a number of new initiatives.

#### Geographical coverage/gradient studies

**Fig. 1:**
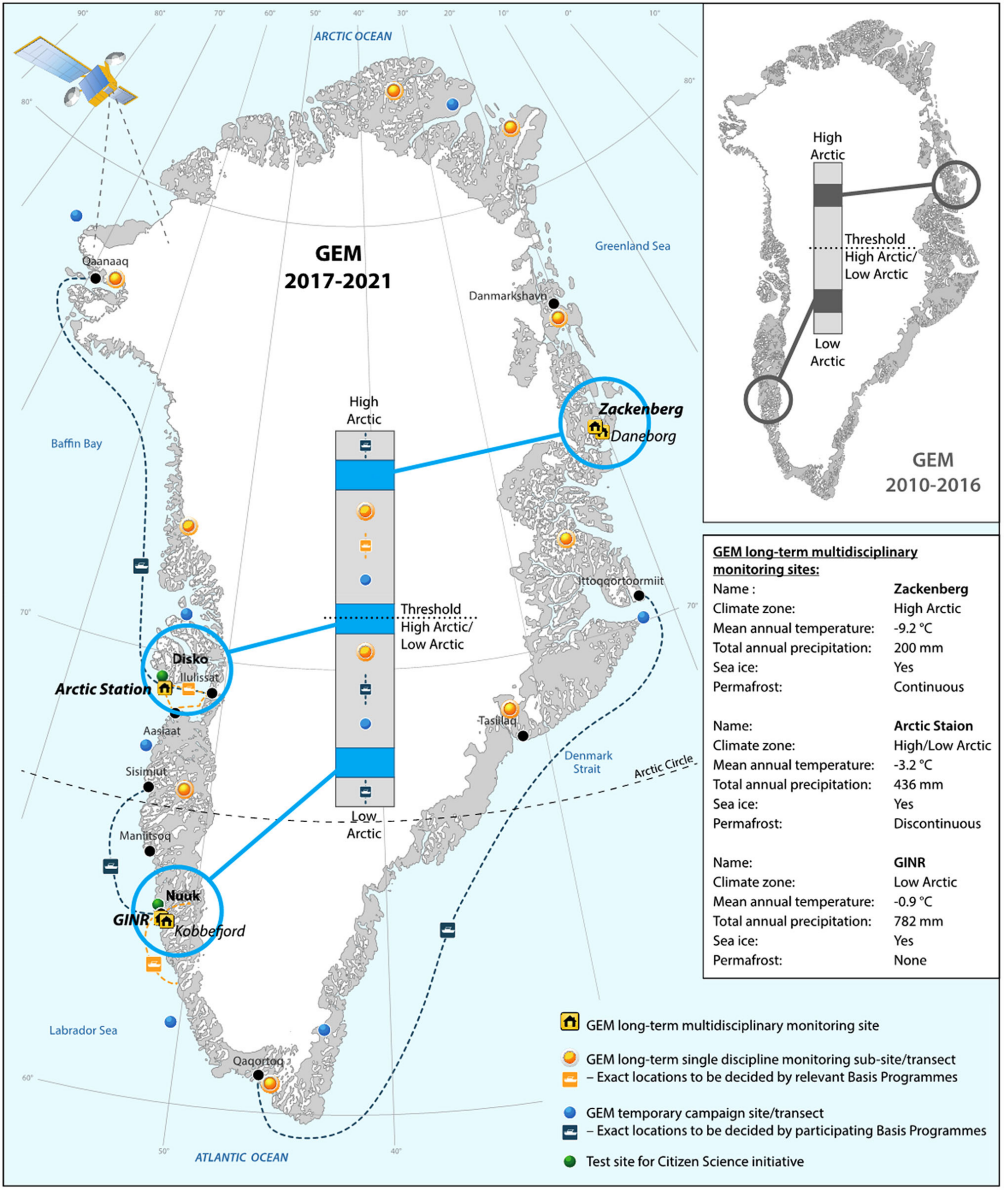
Sampling strategy and geographical coverage of the GEM 2017–2021 strategy period. The location of long-term single discipline sites/transects, temporary/campaign sites/transects and test sites for citizen science are indicative and may change when specific GEM activities are planned (from Christensen and Topp-Jørgensen 2016)

We will aim at including Disko Island with Arctic Station as a long-term multidisciplinary monitoring site for GEM (supplementing existing sites Zackenberg and Nuuk/Kobbefjord), using its location on the boundary between High-Arctic and Low-Arctic to expand the climatic gradient covered by GEM (see Fig. 1). In addition, long-term single disciplinary monitoring sites/transects and short-term campaigns will be integrated to study gradients and enhance process understanding for upscaling and modelling purposes, e.g. climatic measuring stations at selected gradient sites and ship routes.

GEM will also establish an overarching science initiative on remote sensing to support upscaling, prediction and modelling efforts. This will build on existing remote sensing products calibrated and adjusted through ground-based measurements and can be used to upscale key climate and ecosystem parameters.

GEM will furthermore identify and test the potential for citizen science initiatives to contribute to GEM long-term monitoring.

#### Cooperation beyond GEM

GEM will establish closer linkages to Danish/Greenlandic and international institutions/organisation/programmes running extensive/relevant long-term science operations in Greenland and seek to integrate relevant external science projects undertaken at sites of relevance to GEM. While the GEM realm includes glacial, terrestrial, limnic and near coastal processes, linkages to projects covering the ice sheet proper (such as Programme for Monitoring the Greenland Ice Sheet, PROMICE) and the continental shelf (such as surveys by Greenland Institute of Natural Resources) will be sought to link processes from the ice cap to the deep sea, and potentially upscale selected parameters to full Greenlandic scale. GEM will also continue its participation in international scientific fora and contribute to international thematic repositories (e.g. WMO, WHYCOS, CALM, ITEX and WGMS) and assessments (e.g. Arctic Council assessments: AMAP, SWIPA, CBMP and ABA).

### Societal interests

GEM has until now focused primarily on ecosystem mapping and process understanding in a changing climate, but it will in the coming strategy period increase its focus on applied science. There is an expressed interest from government bodies in widening the use of GEM data and results. This includes upscaling results to Greenlandic scale and contribute to national, regional and global networks, programmes and assessments putting Greenland into an Arctic and global perspective.

GEM will also aim to increase its societal relevance through increased focus on linking GEM monitoring activities (e.g. ecosystem functioning, resilience, upscaling and ecosystem services) to societal needs (e.g. authorities and commercial stakeholders) and studying cumulative impacts of climate change and regional development. GEM will strengthen the linkages to relevant high-level climate and ecosystem assessments and initiatives supporting policy and decision making, where GEM data and products provide important contributions (including Greenland Institute of Natural Resources (GINR) and Danish Meteorological Institute).

### Education and outreach

All data generated by GEM are made publicly available.^1^ These data are used by many scientists relating their studies to GEM background data or comparable GEM data series. GEM will also provide data for relevant arctic and global repositories and assessments ensuring a wider use of GEM data. The scientific output of GEM itself will be scientific papers addressing overarching science questions and priorities, with shorter communications in the form of internet-based annual reporting presenting interesting observations or trends of the year. GEM will also support decision making by contributing to the advice of relevant authorities on, for example, ecosystem status and trend, sustainable use of natural resources and cumulative impacts of climate change and regional development.

The GEM programme and monitoring sites provide an ideal platform for educating the next generation of arctic scientists. With freely available data, interdisciplinary teams of scientists and coordinated logistics, GEM will be developed to be more directly applied in an educational context. This will be facilitated and stimulated by the recent establishment of graduate level teaching based in Nuuk at GINR and joint MSc projects, PhD students and post docs between participating institutions.

The GEM studies presented in this special issues of *Ambio* and the many projects and assessments that has used GEM data over recent years contribute to a deeper understanding of climate change and its impact on ecosystem patterns and dynamics in Greenland and the Arctic that form a solid foundation for implementation of the GEM Strategy 2017–2021 as outlined above. It is our hope that this special issue of *Ambio* inspires existing monitoring programmes, scientists and students to contribute to our common knowledge and understanding on how ecosystems respond in a changing Arctic, and what implication this has for the global climate system and local communities, nations, businesses and other stakeholders.

## Footnotes

1
http://data.g-e-m.dk/.
